# Pneumoperitoneum, pneumoretroperitoneum and pneumomediastinum: rare complications of perforation peritonitis: a case report

**DOI:** 10.1186/s13256-024-04488-1

**Published:** 2024-04-17

**Authors:** H. Hafiani, N. Bouknani, E. M. Choukri, R. Charif Saibari, A. Rami

**Affiliations:** Cheikh Khalifa International Hospital, Mohamed VI University of Health Sciences (UM6SS), Ave Mohamed Taieb Naciri, Casablanca, Morocco

**Keywords:** Pneumomediastinum, Pneumoperitoneum, Pneumoretroperitoneum, Perforation, Perforated, Peritonitis, Diverticulitis, Case report

## Abstract

**Background:**

Gas extravasation complications arising from perforated diverticulitis are common but manifestations such as pneumoperitoneum, pneumoretroperitoneum, and pneumomediastinum happening at the same time are exceedingly rare. This case report explores the unique presentation of these 3 complications occurring simultaneously, their diagnosis and their management, emphasizing the importance of interdisciplinary collaboration for accurate diagnosis and effective management.

**Case presentation:**

A 74-year-old North African female, with a medical history including hypertension, dyslipidemia, type 2 diabetes, goiter, prior cholecystectomy, and bilateral total knee replacement, presented with sudden-onset pelvic pain, chronic constipation, and rectal bleeding. Clinical examination revealed hemodynamic instability, hypoxemia, and diffuse tenderness. After appropriate fluid resuscitation with norepinephrine and saline serum, the patient was stable enough to undergo computed tomography scan. Emergency computed tomography scan confirmed perforated diverticulitis at the rectosigmoid junction, accompanied by the unprecedented presence of pneumoperitoneum, pneumoretroperitoneum, and pneumomediastinum. The patient underwent prompt surgical intervention with colo-rectal resection and a Hartmann colostomy. The postoperative course was favorable, leading to discharge one week after admission.

**Conclusions:**

This case report highlights the clinical novelty of gas extravasation complications in perforated diverticulitis. The unique triad of pneumoperitoneum, pneumoretroperitoneum, and pneumomediastinum in a 74-year-old female underscores the diagnostic challenges and the importance of advanced imaging techniques. The successful collaboration between radiologists and surgeons facilitated a timely and accurate diagnosis, enabling a minimally invasive surgical approach. This case contributes to the understanding of atypical presentations of diverticulitis and emphasizes the significance of interdisciplinary teamwork in managing such rare manifestations.

**Supplementary Information:**

The online version contains supplementary material available at 10.1186/s13256-024-04488-1.

## Introduction

Potential sources of gas extravasation include the respiratory tract, the gastrointestinal tract or infections with gas-generating germs [1]. While pneumoperitoneum is a classic complication of diverticulitis, pneumomediastinum [2] and pneumoretroperitoneum are very rare complications of perforated diverticulitis [3]. Imaging studies can help to diagnose such diseases, their complications and even sometimes, their own etiology. While abdominal X-ray alone can help diagnose air outside the peritoneum, CT scan remains the gold standard today with fine localisation of air bubbles, eventual ascites and other things such as perforation location. We present the unusual case of a 74 years old female with peritonitis from perforated diverticulitis at the rectosigmoid junction that resulted in pneumoperitoneum, pneumoretroperitoneum and even pneumomediastinum.

## Case presentation

The patient of the case is a 74 years old North African female with hypertension, dyslipidemia, type 2 diabetes, goiter, prior cholecystectomy, and bilateral total knee replacement. The patient's symptoms began with sudden onset of cramp-like pelvic pain, accompanied by chronic constipation and scant rectal bleeding. Notably, there were no associated vomiting or urinary symptoms, but the presentation occurred within a febrile and altered general condition.

**Fig. 1:**
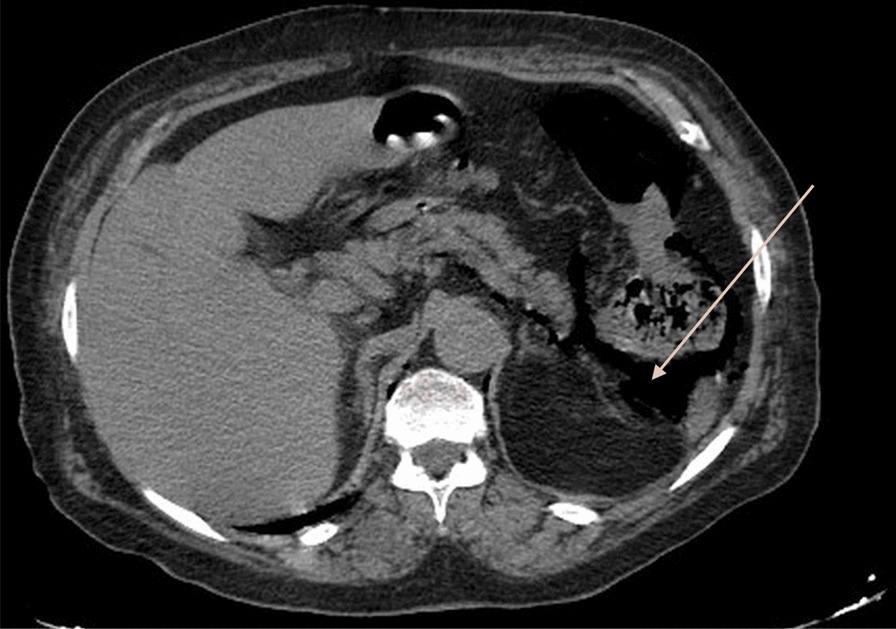
CT scan axial view showing pneumoperitoneum. Arrow points to pneumoperitoneum

**Fig. 2:**
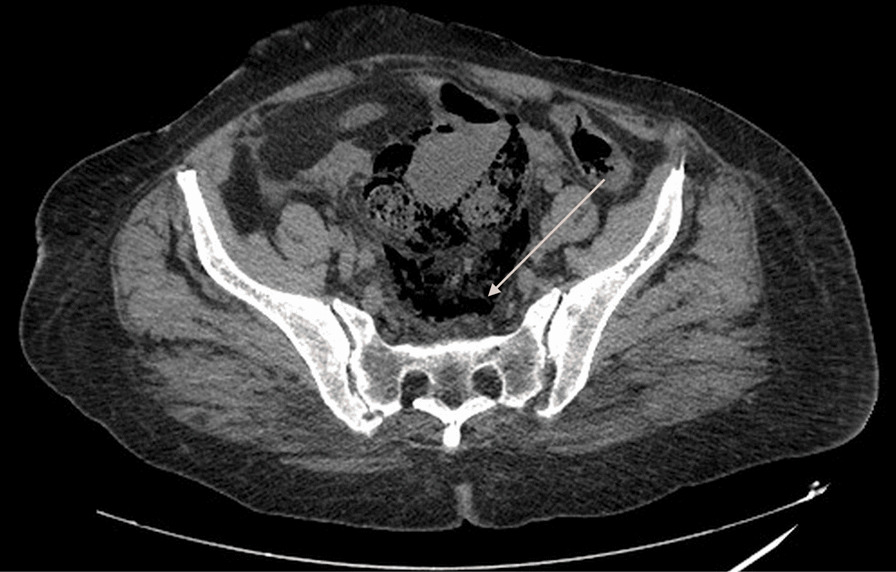
CT scan axial view showing pneumoretroperitoneum. Arrow points to pneumoretroperitoneum

**Fig. 3:**
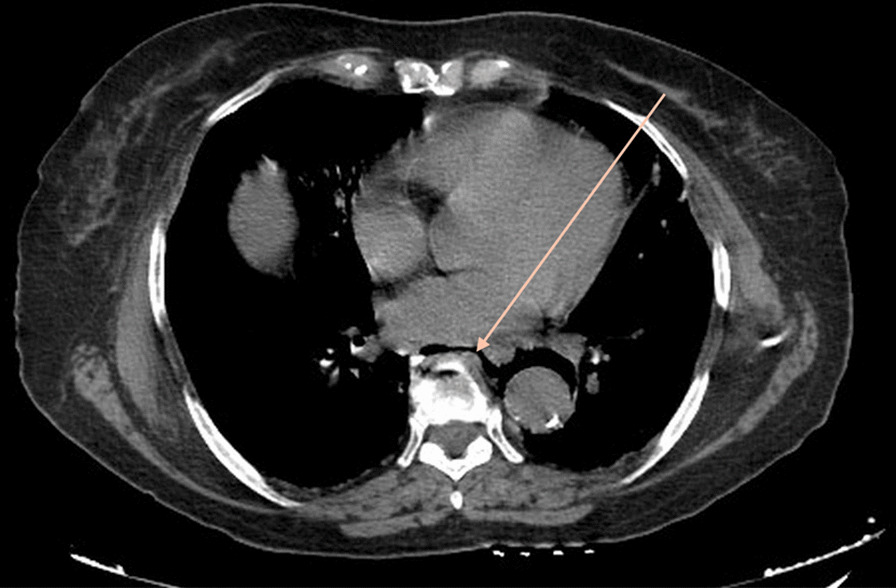
CT scan axial view showing pneumomediastinum. Arrow points to pneumodiastinum

Clinical examination showed hemodynamic and respiratory instability with low blood pressure and hypoxemia associated with diffuse tenderness and hypogastric guarding, while rectal examination didn’t show any rectal bleeding or melena. After appropriate resuscitation done with appropriate quantities of norepinephrine and saline serum, the patient was stable enough to undergo imaging. A CT scan was ordered at the emergency room and the final diagnosis was perforated diverticulitis but what caught our attention was that the patient had both pneumoperitoneum (Fig. 1) and pneumoretroperitoneum (Fig. 2) and pneumomediastinum (Fig. 3) that suggested perforation at the rectosigmoid junction.

Our patient was sent to the operating room for surgery on that same day and had laparoscopic colo-rectal resection with a Hartmann colostomy. The postoperative course was favorable and the patient was discharged from the hospital 1 week afterward.

## Discussion

Perforation of the colic wall can happen due to diverticulitis, neoplasm, iatrogenic or traumatic mechanisms. Colonic diverticulosis is common in the western countries affecting nearly 50% of the population [4] with approximately 20% of them that may develop inflammation of the diverticula [5]. This inflammation can lead to perforation which is a serious complication that requires urgent intervention. Extradigestive air secondary to perforated diverticula can help localize the site of the perforation on CT scan, whether it is in the peritoneum, behind it, or in the mediastinum. While pneumoperitoneum is a classic localisation of air after perforation, pneumoretroperitoneum is less usual.

Pneumomediastinum secondary to colonic perforation is extremely rare and only 20 cases of spontaneous perforation (not iatrogenic or traumatic) were reported before 2019 [6]. Diverticulitis was the most common cause of mediastinal emphysema [6].

**Fig. 4:**
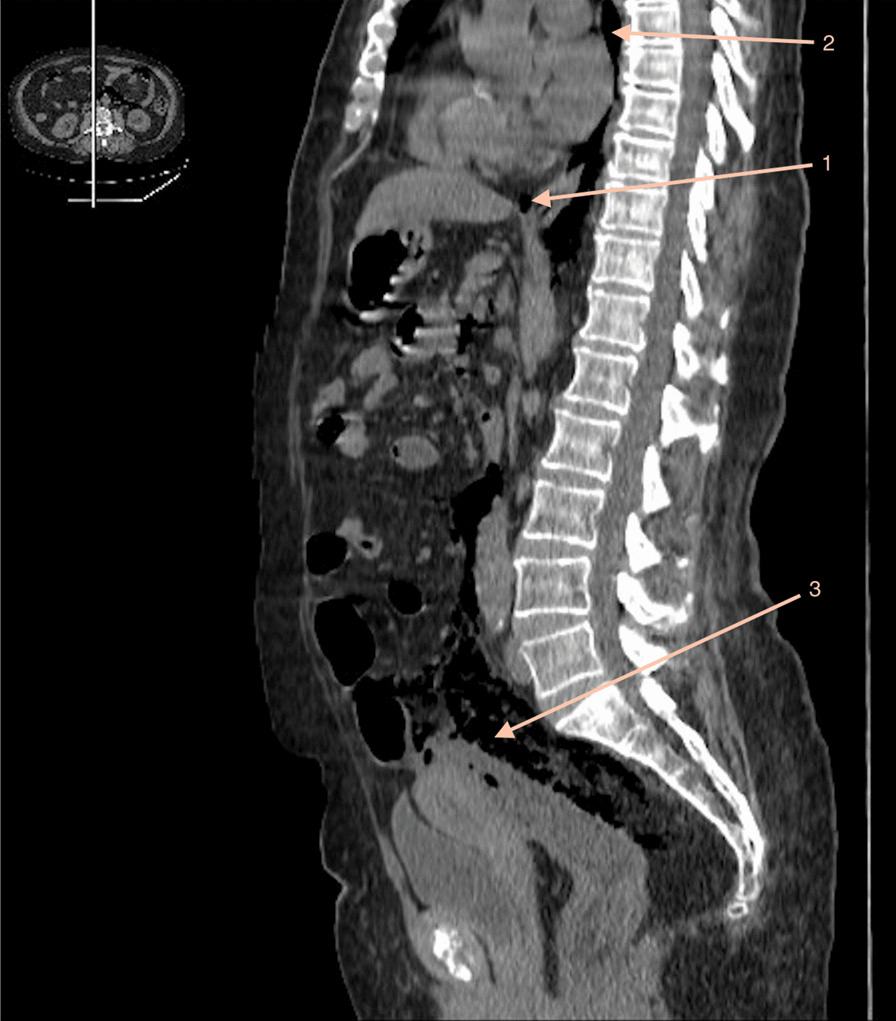
CT scan sagittal view showing pneumoperitoneum [1], pneumoretroperitoneum [3] and pneumomediastinum [2]. Arrow 1 points to pneumoperitoneum, arrow 2 points to pneumomediastinum, arow 3 points to pneumoretroperitoneum

In our case, the air was localized in the 3 parts (Fig. 4) and made us immediately think that the perforation occurred at the rectosigmoid junction, near the Douglas, where the peritoneum folds (Fig. 2). The mechanism of the pneumomediastinum is not fully understood but a few theories emerged: it could either come from extravasation of air through the fascial planes or the esophagus and its perivascular spaces or come directly from the retroperitoneum [7].

Another theory includes the foramina of Morgagni and Bochdalek, which are responsible for diaphragmatic hernias when they are weak. These 2 visceral peritoneal folds could constitute air passage from the peritoneum to the mediastinum.

In our particular scenario, the radiologist readily established the diagnosis due to clear manifestations of diverticulitis in addition to the presence of extradigestive air. However, in certain instances, the detection of air may serve as the sole indicator, necessitating extensive paraclinical investigations. This underscores the rationale behind the diagnostic algorithm proposed by Wang et al. [8] for situations where air constitutes the sole available information.

Following the diagnosis, the patient promptly underwent laparoscopic colorectal resection, during which the surgeons validated the radiologist's diagnosis of peritonitis resulting from diverticulitis perforation (Additional file 1: Video S1). Peritoneal lavage was done, and a Hartmann colostomy was performed by the surgeon. Subsequently, the patient was discharged without any complications after a 10-day hospitalization period.

The question of the origin of the extradigestive air remains, and this case highlights the fact that the collaboration between radiologists and surgeons should be optimal. With a good and clear diagnosis, the surgeon chose the laparoscopic approach (less harmful for the patient) and could cure a potentially fatal disease with a minimalist approach, sending the patient back home 10 days after admission.

## Conclusion

In conclusion, our case report underscores the complexity and rarity of gas extravasation complications resulting from perforated diverticulitis. The presentation of a 74-year-old female with peritonitis at the rectosigmoid junction led to the unique occurrence of pneumoperitoneum, pneumoretroperitoneum, and pneumomediastinum. This very unusual manifestation necessitated a prompt and collaborative effort between radiologists and surgeons for accurate diagnosis and timely intervention. The effective coordination between radiologists and surgeons, coupled with advanced imaging techniques, not only facilitated a timely and accurate diagnosis but also enabled a minimally invasive surgical approach with a favorable outcome.

### Supplementary Information

**Additional file 1.** Video of the laparoscopic surgery showing the perforation.

## Data Availability

The data that support the findings of this study are available from the corresponding author, Hafiani Hamza, upon reasonable request.
